# Modeling of CO_2_-LPG WAG with asphaltene deposition to predict coupled enhanced oil recovery and storage performance

**DOI:** 10.1038/s41598-021-81316-2

**Published:** 2021-03-02

**Authors:** Jinhyung Cho, Baehyun Min, Moon Sik Jeong, Young Woo Lee, Kun Sang Lee

**Affiliations:** 1 Center for Climate/Environment Change Prediction Research, Ewha Womans University, 52 Ewhayeodae-gil, Seodaemun-gu, Seoul, 03760 Republic of Korea; 2 Department of Climate and Energy Systems Engineering, Ewha Womans University, 52 Ewhayeodae-gil, Seodaemun-gu, Seoul, 03760 Republic of Korea; 3 Department of Earth Resources and Environmental Engineering, Hanyang University, 222 Wangsimni-ro, Seongdong-gu, Seoul, 04763 Republic of Korea

**Keywords:** Climate-change mitigation, Carbon capture and storage, Crude oil

## Abstract

Combined carbon capture and storage and CO_2_-enhanced oil recovery (CCS-EOR) can reconcile the demands of business with the need to mitigate the effects of climate change. To improve the performance of CCS-EOR, liquefied petroleum gas (LPG) can be co-injected with CO_2_, leading to a reduction in the minimum miscibility pressure. However, gas injection can cause asphaltene problems, which undermines EOR and CCS performances simultaneously. Here, we systematically examine the mechanisms of asphaltene deposition using compositional simulations during CO_2_-LPG–comprehensive water–alternating-gas (WAG) injection. The LPG accelerates asphaltene deposition, reducing gas mobility, and increases the performance of residual trapping by 9.2% compared with CO_2_ WAG. In contrast, solubility trapping performance declines by only 3.7% because of the greater reservoir pressure caused by the increased formation damage. Adding LPG enhances oil recovery by 11% and improves total CCS performance by 9.1% compared with CO_2_ WAG. Based on reservoir simulations performed with different LPG concentrations and WAG ratios, we confirmed that the performance improvement of CCS-EOR associated with increasing LPG and water injection reaches a plateau. An economic evaluation based on the price of LPG should be carried out to ensure practical success.

## Introduction

Concerns about climate change are driving global efforts to reduce atmospheric CO_2_ concentrations. Many options to reduce CO_2_ emissions have been studied. Climate change mitigation will depend at least in part on combining CO_2_ capture and utilization with microalgal carbon capture and biomass production^1^. Improving energy efficiency and using renewable energy in residential and industrial contexts can also reduce CO_2_ emissions. Carbon capture and storage (CCS) is regarded as an effective way to reduce atmospheric CO_2_ concentrations due to its negative CO_2_ emissions^2,3^. Forty-three large-scale commercial CCS facilities were in operation globally in 2018^4^, with that number increasing to 51 in 2019^5^. These operations have the combined capacity to capture and store an estimated 40 million tons of CO_2_ every year. The disadvantage of CCS in aquifers is the lack of a profitability model. Coupling CO_2_-enhanced oil recovery with CCS (CCS-EOR) could improve the economics through oil production^6,7^.

Miscible gas flooding as an EOR technology changes the composition of oils and results in the precipitation and deposition of asphaltene in oil reservoirs^8^ as changes in the composition of the oil affect the solubility of asphaltene^9,10^. Precipitated asphaltenes can be deposited throughout a reservoir, damaging geological formations. Such damage can affect both EOR performance and CO_2_ storage mechanisms during CCS-EOR^11^. Oil and asphaltene do not have to be taken into account in conventional CCS involving aquifers. But in CCS-EOR, both the oil and asphaltene phases must be considered to enable precise performance predictions due to interactions among phases.

The effectiveness of CO_2_ injection in CCS-EOR is limited due to difficulties reaching miscible conditions, which requires that the reservoir pressure exceed the minimum miscible pressure (MMP). Increasing only the injection pressure is challenging because of the cost and the risk of formation damage. Co-injection of liquefied petroleum gas (LPG) and CO_2_ can address this problem by lowering the MMP instead of increasing reservoir pressure^12,13^. CO_2_-LPG co-injection improves both the displacement and vertical-sweep efficiencies of EOR, but its effectiveness depends on other factors, such as heterogeneity and reservoir fluid properties^14,15^. Experimental studies of CO_2_-LPG flooding have verified the advantages of LPG^12,13^. Because adding LPG accelerates oil swelling by improving miscibility and reducing oil density, it also intensifies asphaltene precipitation^16^. Previous studies assumed asphaltene only affected EOR, and not CO_2_ storage^17,18^. To overcome these application limits, an integrative model that considers asphaltene deposition in a CO_2_-LPG system was developed for this study. The model is an improved version of a CCS-EOR model developed by Cho et al.^11^ that used only CO_2_ injection. The new model can take into account the effects of both CO_2_-LPG co-injection and formation damage on CO_2_-trapping mechanisms during CCS-EOR.

### Numerical model for major mechanisms

#### Porosity and permeability reduction

A solid model proposed by Gupta^19^ was used for this study. Precipitating and non-precipitating components were used to calculate the fugacity of the solid phase^20^. While the solid model calculates the fugacity of the precipitating asphaltene, *S*_1_, using Eq. (1), the Peng-Robinson equation of state (EOS)^21,22^ computes the fugacity of the components in oil and gas phases:

1 $$\ln f_{{S_{1} }} = \ln f_{{S_{1} }}^{*} + \frac{{V_{{S_{1} }} \left( {p - p^{*} } \right)}}{RT}$$

where $f_{{S_{1} }}^{*}$ is the fugacity of *S*_1_ at onset pressure p^*^ and $V_{{S_{1} }}$ is the asphaltene molar volume. The equilibrium condition can be achieved when the fugacities of each component in solid (the solid model), water (Henry’s law), oil, and gas (Peng-Robinson EOS) are equal. The amount of asphaltene precipitation can be estimated using the equilibrium condition.

After estimating asphaltene precipitation through the equilibrium condition of the fugacity of each component, the Wang and Civan^23^ equation can be applied to estimate the amount of asphaltene deposition as follows:

2 $$\frac{{V_{{S_{2}^{d} }}^{n + 1} - V_{{S_{2}^{d} }}^{n} }}{\Delta t} = \alpha C_{{S_{2}^{f} }}^{n + 1} \phi^{n + 1} - \beta V_{{S_{2}^{d} }}^{n + 1} \left( {v_{o}^{n} - v_{cr,o} } \right) + \gamma u_{o}^{n} C_{{S_{2}^{f} }}^{n + 1}$$

where $V_{{S_{2}^{d} }}$ and $C_{{S_{2}^{f} }}$ are the volume of deposited and flowing solid *S*_2_ and *v*_*o*_ and *v*_*cr*,*o*_ are the interstitial and critical velocity of the oil phase, respectively. The coefficient of the surface deposition is represented by *α*. The re-entrainment term *β* has a non-zero value when *v*_*o*_ is greater than *v*_*cr*,*o*_ for the re-entrainment phenomenon, and can reduce the amount of asphaltene deposition. If *v*_*o*_ is lower than *v*_*cr*,*o*_, *β* is set to zero and re-entrainment does not occur. The *γ* value is the pore throat-plugging coefficient.

Deposited asphaltene plug pores, which can reduce porosity and absolute permeability, are described by equations for the power law and the oil resistance factor^24^:

3 $$\phi = \phi_{0} - V_{{S_{2}^{d} }}$$

4 $$\frac{{k_{0} }}{k} = \left( {\frac{{\phi_{0} }}{\phi }} \right)^{n} = F_{r,o}$$

where the superscript 0 refers to the initial condition and *F*_*r*,*o*_ is the oil resistance factor.

### Residual and solubility trapping

Asphaltene deposition not only changes absolute permeability but also relative permeability with wettability alterations^25^. To reflect this effect, the Brooks–Corey model is applied with the contact angle change^26^:

5 $$k_{r,w} = k_{r,w}^{*} \left( {\frac{{S_{w} - S_{r,w} }}{{1 - S_{r,w} - S_{r,nw} }}} \right)^{{e_{w} }}$$

6 $$k_{r,w}^{*} = 1 + \left[ {k_{r,w}^{0*} + \frac{{\cos \theta - \cos \theta^{0} }}{{\cos \left( {\pi - \theta^{0} } \right) - \cos \theta^{0} }}\left( {k_{r,nw}^{0*} - k_{r,w}^{0*} } \right) - 1} \right]\left( {\frac{{1 + T_{w}^{0} N_{T,w}^{0} }}{{1 + T_{w} N_{T,w} }}} \right)$$

7 $$e_{w} = 1 + \left[ {e_{w}^{0} + \frac{{\cos \theta - \cos \theta^{0} }}{{\cos \left( {\pi - \theta^{0} } \right) - \cos \theta^{0} }}\left( {e_{nw}^{0} - e_{w}^{0} } \right) - 1} \right]\left( {\frac{{1 + T_{w}^{0} N_{T,w}^{0} }}{{1 + T_{w} N_{T,w} }}} \right)$$

where subscript *O* indicates the initial condition, and *w* and *nw* indicate the wetting and non-wetting phases, respectively, $k_{r}^{ * }$ is the endpoint of the relative permeability, *S*_*r*_ is the residual saturation, *e*_*w*_ is the exponential parameter, and *T*_*w*_ and *N*_*T*_ are the trapping parameter and number, respectively.

**Figure 1:**
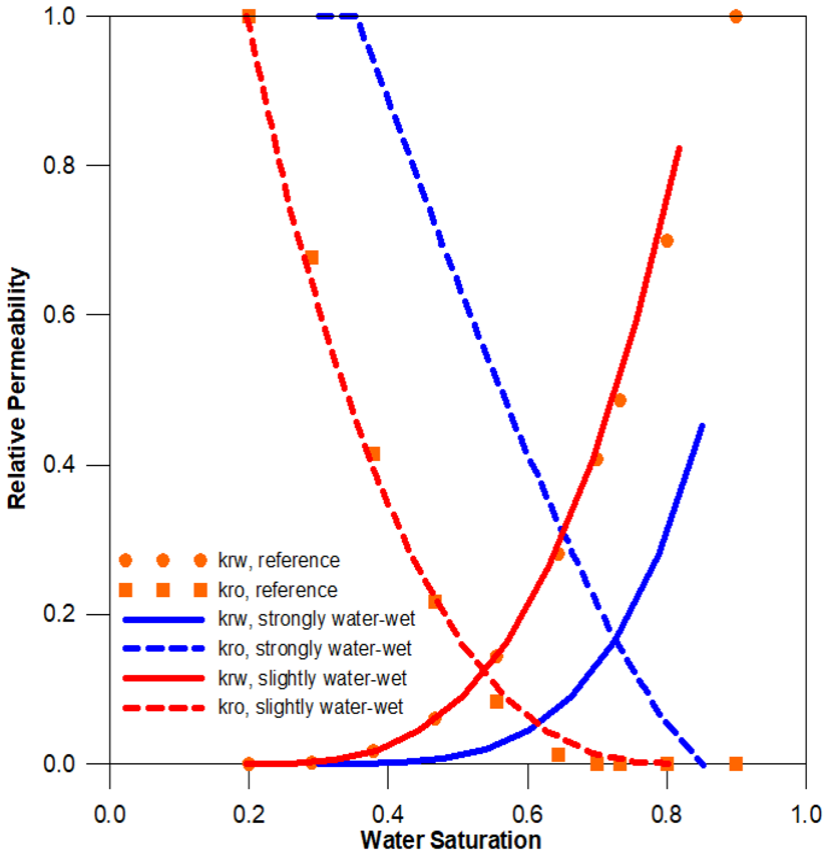
Water and oil relative permeability curves^11^.

Relative permeability and contact angle changes are assumed to be affected only by asphaltene deposition. The initial condition of reservoir is considered as strongly water-wet, changing to slightly water-wet at maximum asphaltene deposition, as determined by the interpolation method. To ensure reliability, the same reference is used for relative permeability and fluid data, which are widely used in asphaltene simulation studies, and a previous base study^11,27^. The reservoir gradually changes to slightly water-wet conditions using the interpolation method, depending on the amount of asphaltene deposition (Fig. 1).

The Larsen and Skauge^28^ three-phase hysteresis model can be applied to reflect the residual trapping performance with the estimated relative permeability curves. Aziz and Settari^29^ derived oil’s relative permeability in a three-phase system from two-phase relative permeability data through a modified version of Stone’s first model. Henry’s law^30^ estimates solubility-trapping performance. While an EOS calculates the fugacity of CO_2_ in gas and oil phases, Henry’s law calculates fugacity in the aqueous phase.

## Results

### Asphaltene and fluid modeling

In this study, fluid modeling for asphaltene precipitation and compositional simulations used WinProp and GEM, which were developed by the Computer Modelling Group Ltd. (CMG) in Canada^31,32^.

Many simulation studies have used data from Burke Oil 1^33^, which were also applied here^11,17^. Table 1 provides the oil composition and properties, and the experimental and calculated oil properties produced by a regression method are compared in Table 2, which shows the acceptable results. The MMP of Burke Oil 1, as estimated by the multiple-mixing-cell method, was 33,584 kPa with pure CO_2_^34^. The MacLeod–Sugden correlation^35^ was used to calculate the interfacial tension (IFT) between CO_2_-LPG gas and crude oil. The MMP is considered when the IFT is less than 0.001 mN/m, and we used the same criterion in our study. Results of the MMP computation are shown in Table 3. The higher the LPG (63% propane and 37% butane) concentration, the lower the MMP, a trend that has been verified by previous studies^36,37^.

**Table 1:** Burke Oil 1 composition and properties.

	Composition	*p*_*c*_ (kPa)	*T*_*c*_ (K)	Molecular weight	Acentric factor
CO_2_	0.0246	7376.5	304	44.0	0.225
N_2_	0.0057	3394.4	126	28.0	0.040
CH_4_	0.3637	4600.2	191	16.0	0.008
C_2_H_6_	0.0347	4883.9	305	30.1	0.098
C_3_H_8_	0.0405	4245.5	369	44.1	0.152
IC_4_	0.0059	3647.7	408	58.1	0.176
NC_4_	0.0134	3799.7	425	58.1	0.193
IC_5_	0.0074	3384.3	460	72.2	0.227
NC_5_	0.0083	3374.1	469	72.2	0.251
FC_6_	0.0162	3293.1	508	86.0	0.275
C_7–15_	0.1966	2624.3	653	153.4	0.452
C_16–25_	0.1255	1621.2	810	293.3	0.789
C_26–30_	0.0400	1226.0	899	389.5	1.015
C_31A+_	0.0742	689.01	1076	665.6	1.423
C_31B+_	0.0433	689.01	1076	665.6	1.423

**Table 2:** Oil properties of Burke Oil 1 data and the computed model.

Parameter	Oil 1	Computed model
Saturation pressure (kPa)	20,339	20,340
Molecular weight (g/mol)	171	174
API (°)	19	19

**Table 3:** MMP calculation results based on the multiple-mixing-cell and IFT methods (kPa).

Component	Multiple-mixing cell method	IFT method
CO_2_	33,584	33,000
CO_2_ 90% + LPG 10%	30,681	29,000
CO_2_ 80% + LPG 20%	23,194	24,000

**Figure 2:**
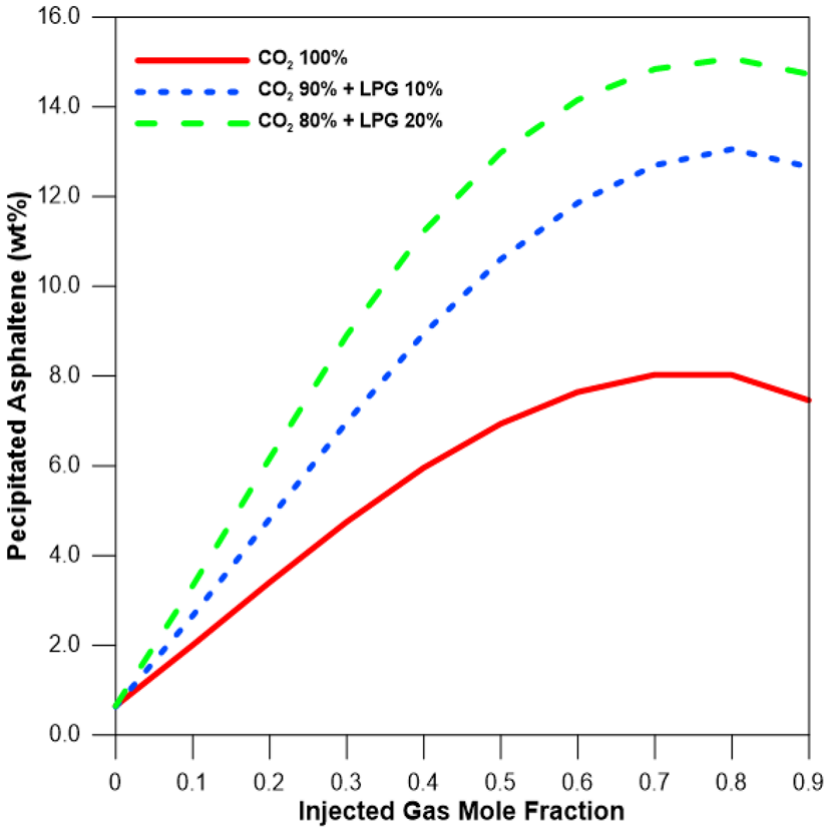
Amount of asphaltene precipitation with various CO_2_ and LPG concentrations at a reservoir pressure of 15,857 kPa and a temperature of 100 °C.

Figure 2 depicts the amount of precipitated asphaltene versus CO_2_ and LPG concentrations at a reservoir pressure of 15,857 kPa. The amount of precipitated asphaltene initially increased with the CO_2_ mole fraction, but it began to decline after the injected gas mole fraction reached 0.7, likely because the saturation pressure also increased with CO_2_ injection, making it a two-phase region^10^. The LPG accelerated more asphaltene precipitation relative to 100% CO_2_ injection^16^.

### EOR performance

Because water–alternating-gas (WAG) injection increases the performance of both EOR and CCS, the process was applied in this study^38,39^. For the first three years, waterflooding was conducted as a secondary recovery, and WAG injection with a 1:1 ratio was performed for 10 years. All input parameters for the simulation are shown in Table 4.

**Table 4:** Simulation input data.

Parameter	Value
Reservoir pressure (kPa)	27,580
Temperature (°C)	100
Permeability (m^2^)	1E^−7^
Porosity (%)	0.25
Initial oil saturation (*S*_*o*_)	0.7
Bottom hole pressure (kPa)	19,300
Total gas injection (PV)	1
*K*_12_	0.1
*K*_21_	0.08
*α*	0.01
*β*	0
*v*_*cr*,*o*_	0
*γ*	0.05
*σ*	150

**Figure 3:**
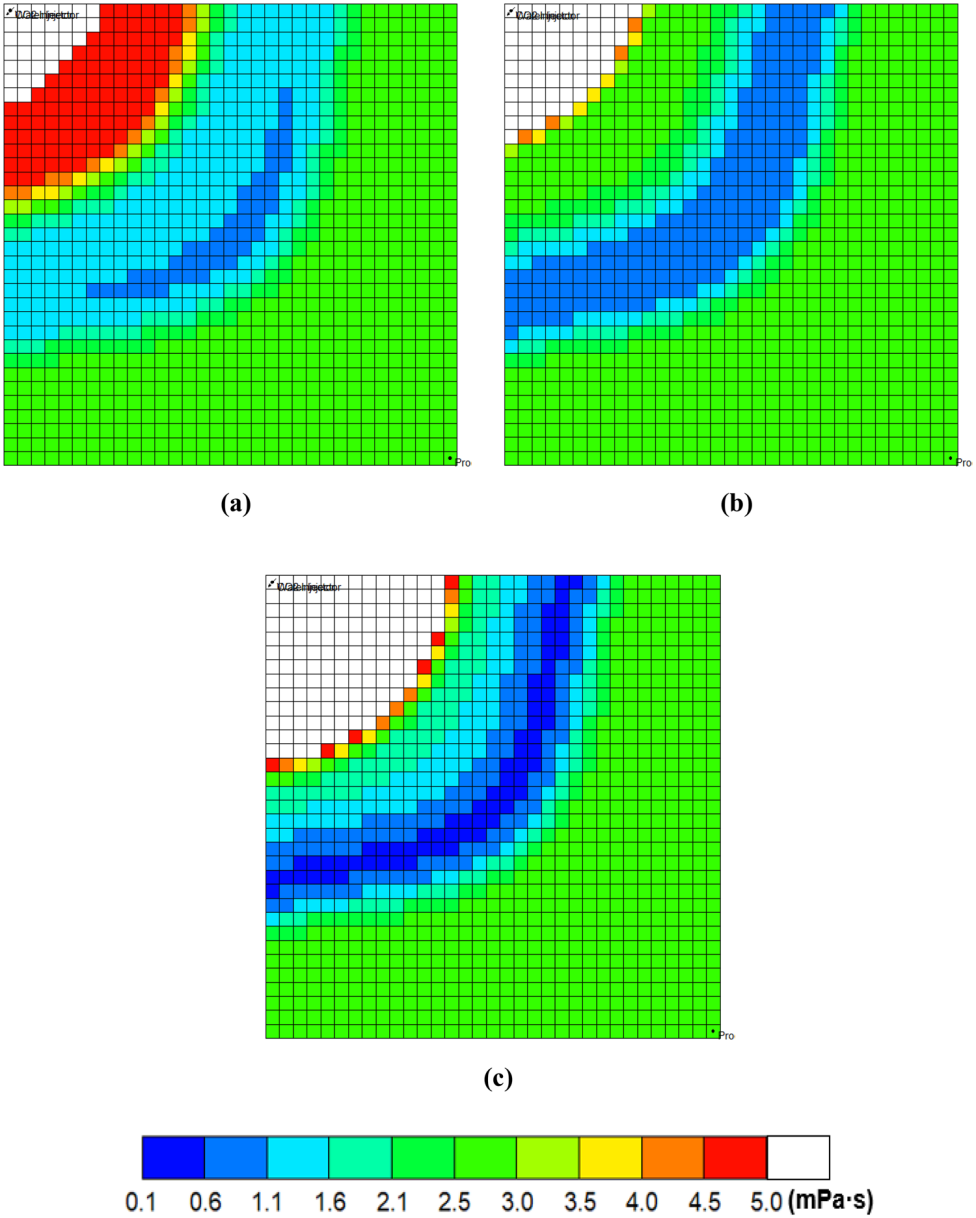
Oil viscosity with WAG: (**a**) 100% CO_2_, (**b**) 90% CO_2_ + 10% LPG, and (**c**) 80% CO_2_ + 20% LPG after the first cycle of gas injection.

**Figure 4:**
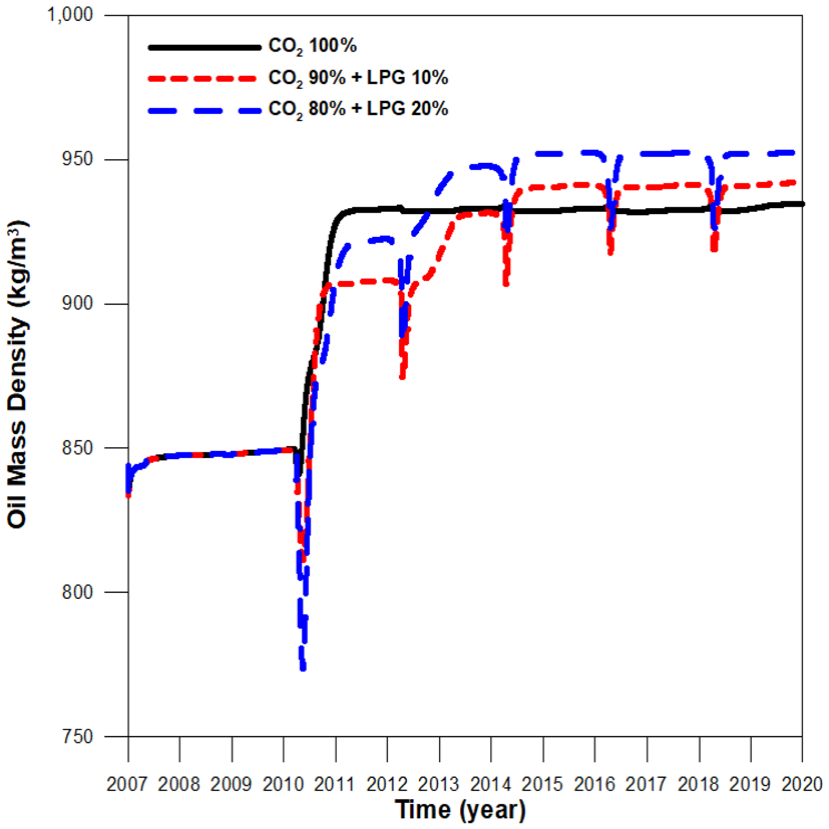
Oil density at the middle of the reservoir during CO_2_ and CO_2_-LPG WAG injection.

Liquid propane gas can increase oil production by improving the displacement efficiency. The added LPG consists of 63% propane and 37% butane^30^. The enhanced displacement efficiency was confirmed by measurements of oil viscosity and density, and IFT changes. Figure 3 depicts the two-dimensional oil viscosity. In the blue region of the swept area where displacing fluids contact reservoir oil the most, the minimum oil viscosity obtained with 100% CO_2_ injection is 1.02 mPa·s (Fig. 3a). When LPG was added as 10% of the molar fraction in the injected gas stream, the molecular weight and viscosity at the reservoir condition were 44.54 and 0.0673 mPa·s, respectively. The CO_2_-LPG associated with the blue region spread more widely, and the oil viscosity decreased to 0.63 mPa·s, which was 38% lower than it in the CO_2_ case (Fig. 3b). As the LPG concentration reached 20%, the oil viscosity decreased to 0.46 mPa·s (Fig. 3c). The white zone, which indicates a viscosity of greater than 5 mPa·s, spreads out near the injector because intermediate components were expelled from the initial oil, and heavier components accounted for a greater percentage of the residual oil. The zone becomes wider with increased LPG concentration due to the improved displacement efficiency. LPG also reduces oil mass density by accelerating oil swelling. Figure 4 illustrates the changes in oil density during WAG injection at the center of the reservoir. The minimum values were 841, 811, and 774 kg/m^3^ for the 100% CO_2_, 90% CO_2_ + 10% LPG, and 80% CO_2_ + 20% LPG cases, respectively, indicating that oil swelling was accelerated by the addition of LPG. In the area swept by the injected gas, oil density began to increase due to heavy components in the residual oil. Residual oil in the 80% CO_2_ + 20% LPG WAG injection had the highest densities, which were 2% and 1% higher than in the 90% CO_2_ + 10% LPG and 100% CO_2_ cases, following the same trend as the residual oil viscosity.

**Figure 5:**
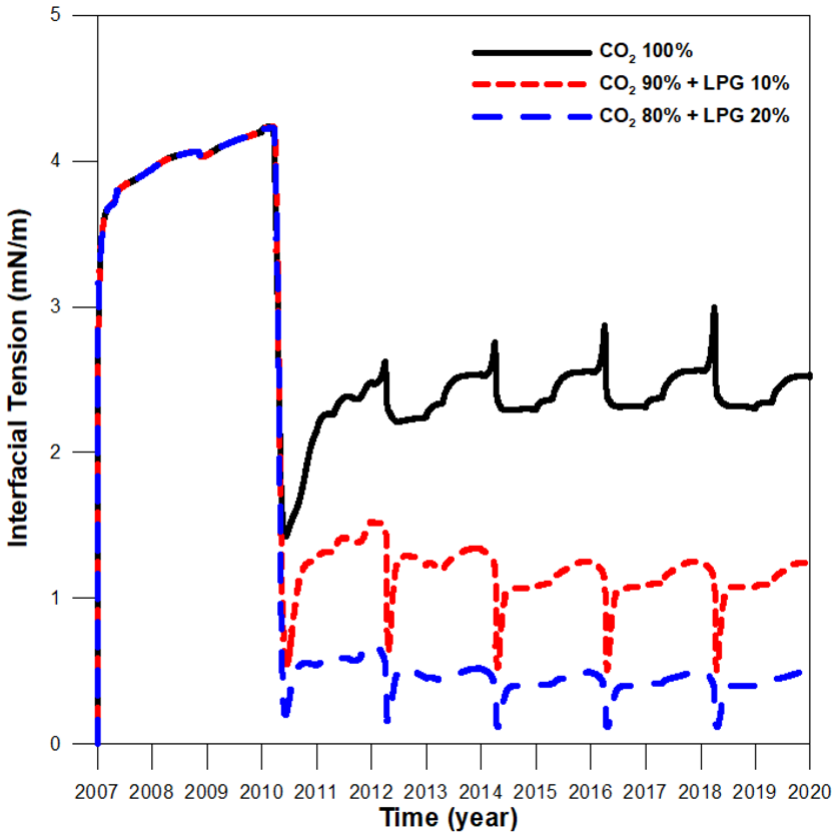
Interfacial tension values between injected CO_2_-LPG gas and the reservoir oil during CO_2_ and CO_2_-LPG WAG injection.

The effect of LPG on the IFT change was investigated using IFT values plotted in the same area (Fig. 5). Because CO_2_ and LPG co-injection increased the density of the displacing fluid (due to the higher molecular weight of LPG), the CO_2_ and LPG mixture became similar to oil, resulting in IFT reduction. As a result, the IFT between the stream with 20% added LPG and the reservoir oil had the lowest value, 0.2 mN/m. The IFT values between the 100% CO_2_ and 90% CO_2_ + 10% LPG streams and the reservoir oil were 1.4 and 0.5 mN/m, which were 600% and 150% higher than that of the stream with 20% added LPG.

**Figure 6:**
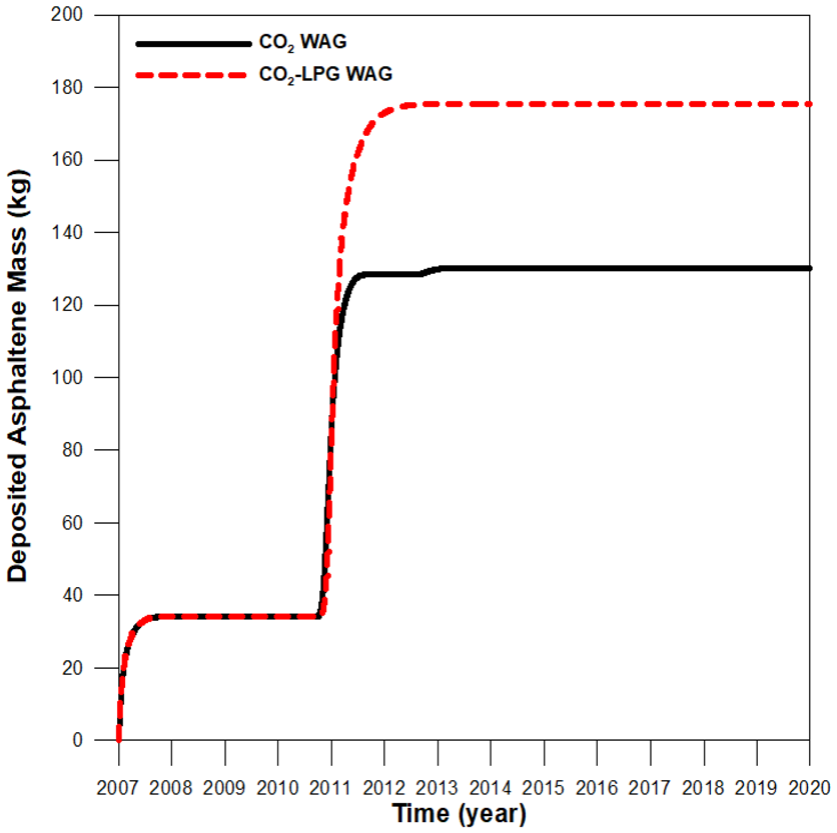
Asphaltene deposition at the middle of the reservoir during CO_2_ and CO_2_-LPG WAG injection.

**Figure 7:**
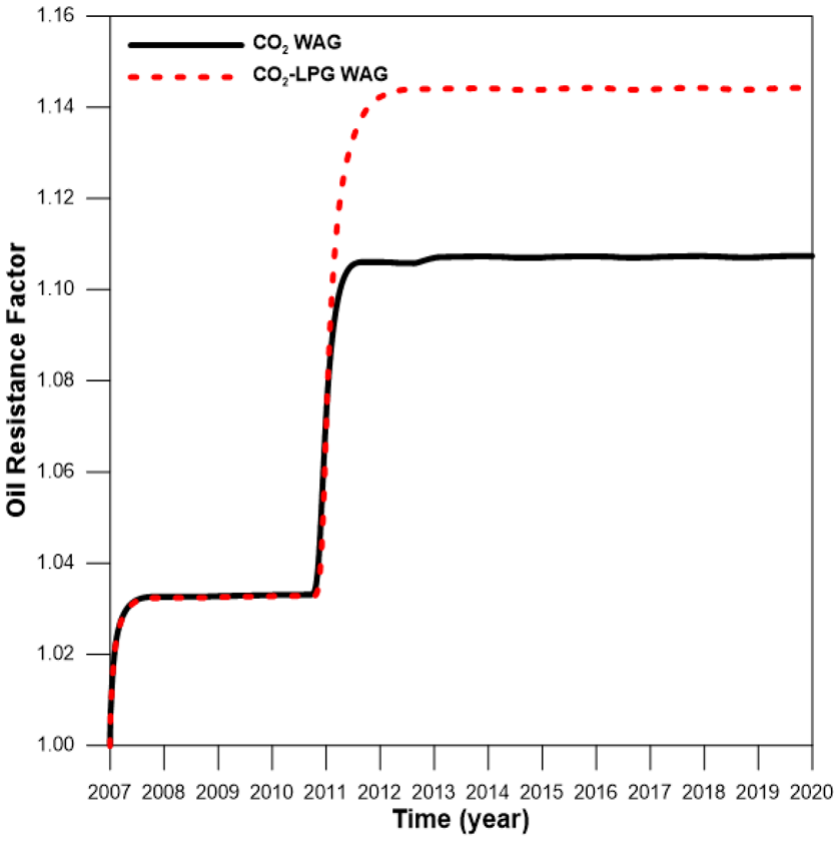
Oil resistance factor during CO_2_ and CO_2_-LPG WAG processes.

**Figure 8:**
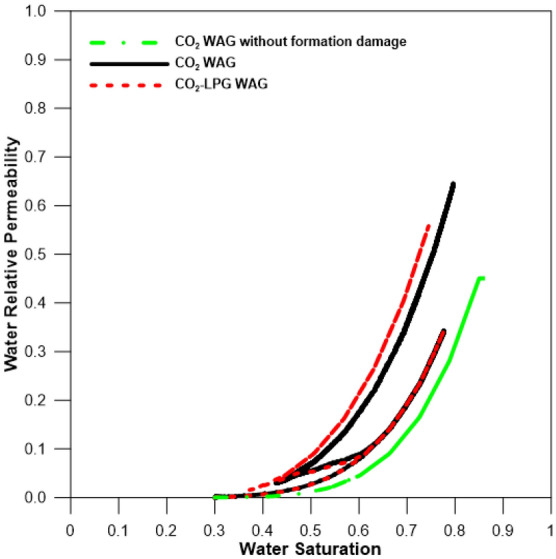
Water relative permeability with and without formation damage during CO_2_ WAG and CO_2_-LPG WAG injection.

**Figure 9:**
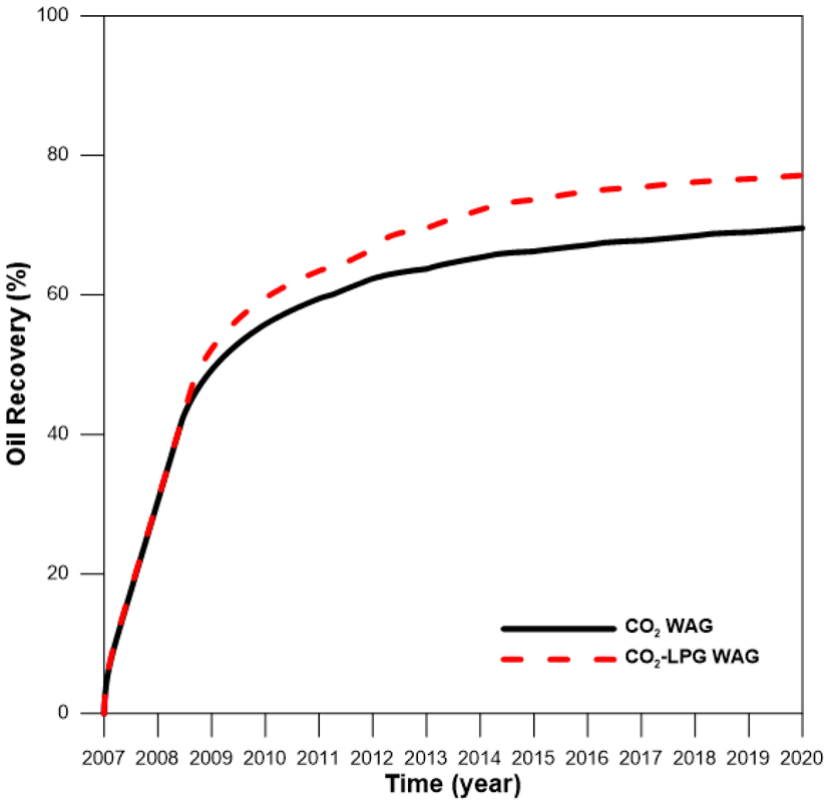
Oil recovery during CO_2_ and CO_2_-LPG WAG injection.

Although adding LPG can improve miscibility, it can also increase asphaltene-related deposition in the reservoir (Fig. 6). The first stage of asphaltene deposition resulted from pressure depletion during pre-waterflooding for three years (2007–2010), and the compositional change of oil caused deposition again after 2010 due to WAG injection. Figure 7 shows the effects of the oil resistance factor, with that of CO_2_-LPG WAG injection 4% higher than it was in CO_2_ WAG injection. Figure 8 supplies the water relative permeability curves after the wettability alteration by asphaltene deposition. Water permeability was enhanced to the water saturation (shown by the black line) after asphaltene deposition during CO_2_ WAG injection. For CO_2_-LPG WAG injection, the improvement was more significant, as indicated by the red line. Overall, LPG improved displacement efficiency, with oil 11% higher than in the case of CO_2_ WAG injection (Fig. 9).

### CCS-EOR performance

**Figure 10:**
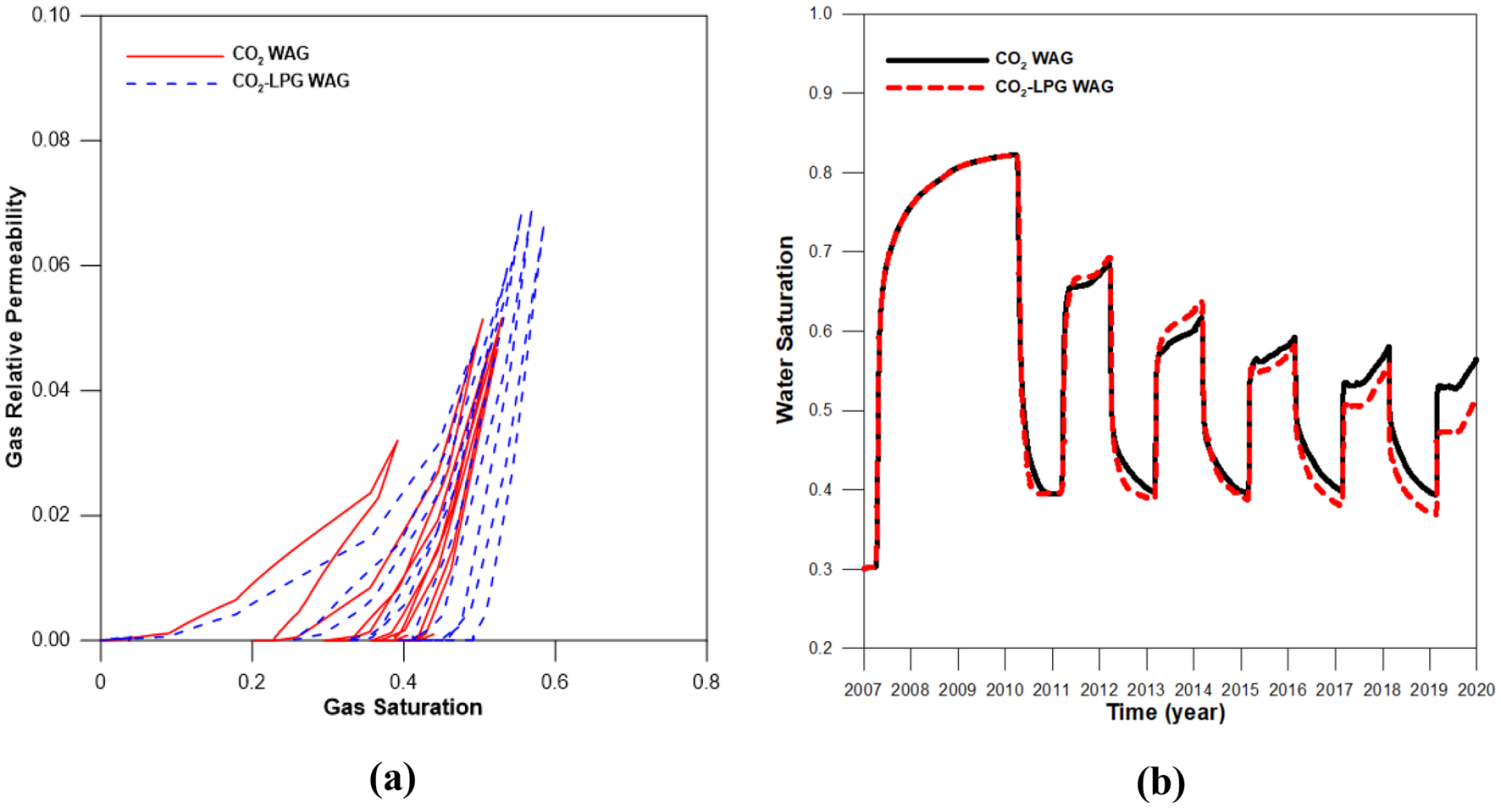
(**a**) Gas relative permeability and (**b**) residual water saturation during CO_2_ and CO_2_-LPG WAG injection.

**Figure 11:**
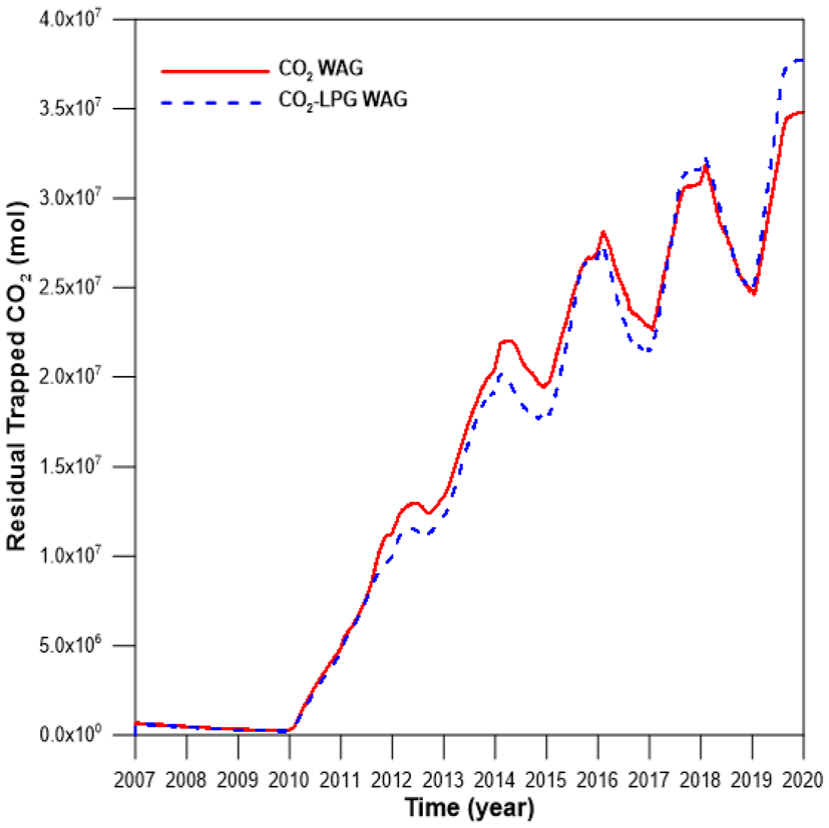
Residual trapped CO_2_ for CO_2_ and CO_2_-LPG WAG models.

**Figure 12:**
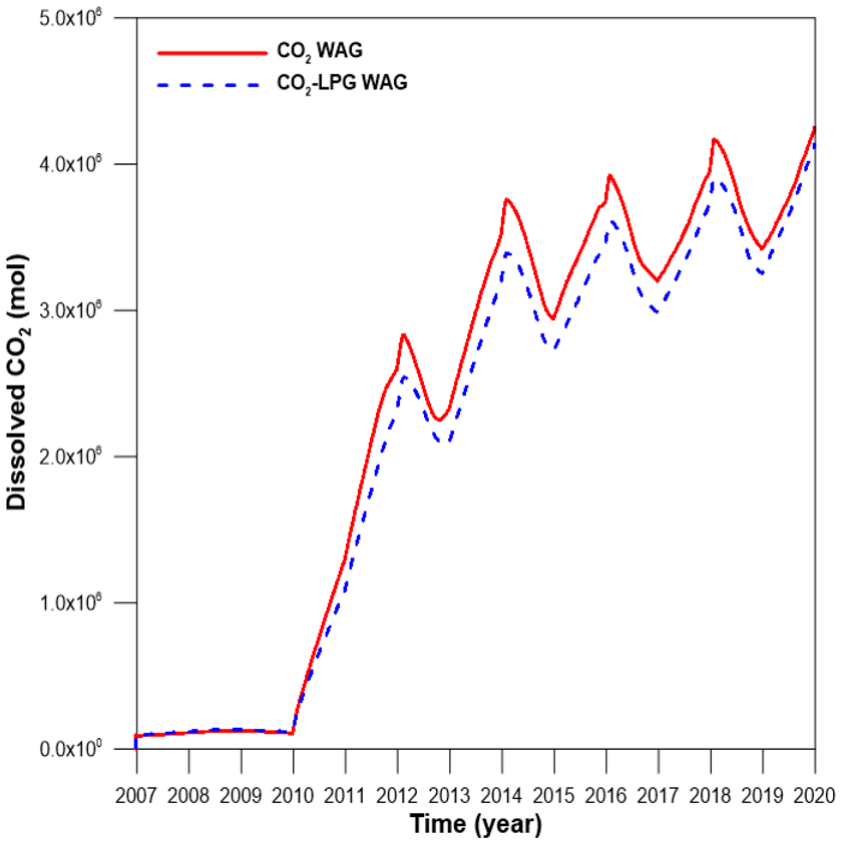
The amount of dissolved CO_2_ for CO_2_ and CO_2_-LPG WAG injection.

**Figure 13:**
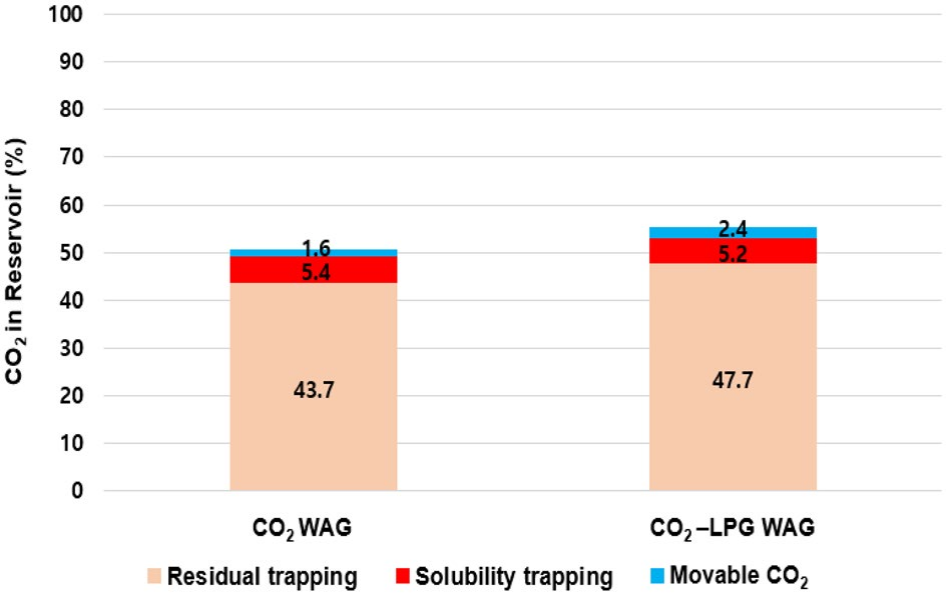
CO_2_ remaining in the reservoir according to trapping mechanism.

To exclude the effects of differences in the injection rates, the same rate was set for injected gas with molar fractions of 100% CO_2_ and 90% CO_2_ + 10% LPG. Figure 10 depicts the gas scanning curves for the asphaltene, hysteresis effects, and residual water saturation. Figure 10a shows the gas relative permeability change during CO_2_ and CO_2_-LPG WAG processes. Because LPG gives the rock surface a higher affinity for oil and gas due to surface deposition of asphaltene, the gas mobility decreased and the relative permeability shifted toward the bottom right. This caused 9% more CO_2_ residual trapping by hysteresis, even though less injected CO_2_ was involved in CO_2_ WAG injection (Fig. 11). In contrast, because LPG accelerated the improvement of water mobility and gas hysteresis, trapped CO_2_ displaced more water, reducing water saturation (Fig. 10b). Consequently, the CO_2_ dissolved in the water in the CO_2_-LPG WAG process was 3.9% lower than in the CO_2_ WAG injection (Fig. 12). However, this does not indicate a reduced performance of CO_2_ solubility trapping. Because the injected moles of CO_2_ were different in this study, the ratio between the remaining and injected CO_2_ in the reservoir should be compared when attempting to make an accurate comparison of trapping performance by mechanism (Fig. 13). The movable CO_2_ was not yet trapped but can be trapped potentially. As shown in Fig. 13, residual trapped and movable CO_2_ were 9.2% and 50% higher than in the CO_2_ WAG case, respectively, while solubility-trapped CO_2_ was 3.7% lower. The addition of LPG caused more wettability alteration, reduced gas mobility, and increased water mobility. Residual trapping performance was improved due to reduced gas mobility. In contrast, solubility trapping decreased because the increased water mobility reduced the residual water saturation. As a result, the total CCS performance was enhanced by 9.1% after the addition of LPG.

### LPG concentration and WAG ratio effects

**Figure 14:**
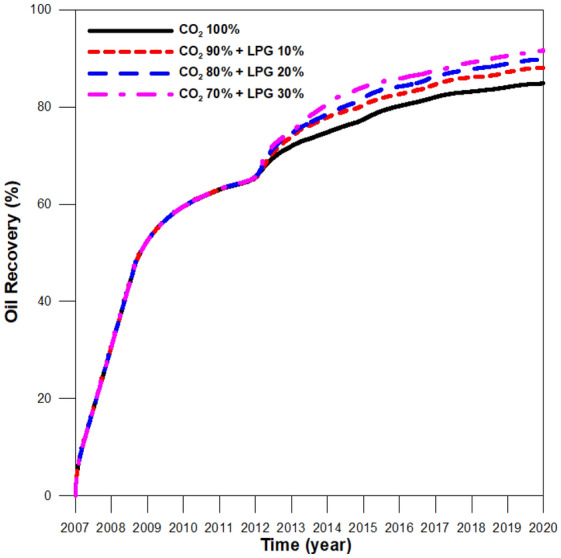
Oil recovery during CO_2_ and CO_2_-LPG WAG injection in integrative models at various LPG concentrations.

By analyzing the effectiveness of adding LPG to the CO_2_ process, we investigated the effects of LPG concentrations and WAG ratios on CCS-EOR performance during CO_2_-LPG WAG injection. As can be seen in Fig. 14, adding 10% LPG to the injection gas led to a 4% increase in oil recovery compared with 100% CO_2_ injection. The recovery of 80% CO_2_ + 20% LPG was 2% higher than that of the 90% CO_2_ + 10% LPG case, and the increase was the same in the 30% LPG + 20% LPG conditions. This indicates that the incremental increase in EOR performance plateaus as the amount of added LPG increases.

**Figure 15:**
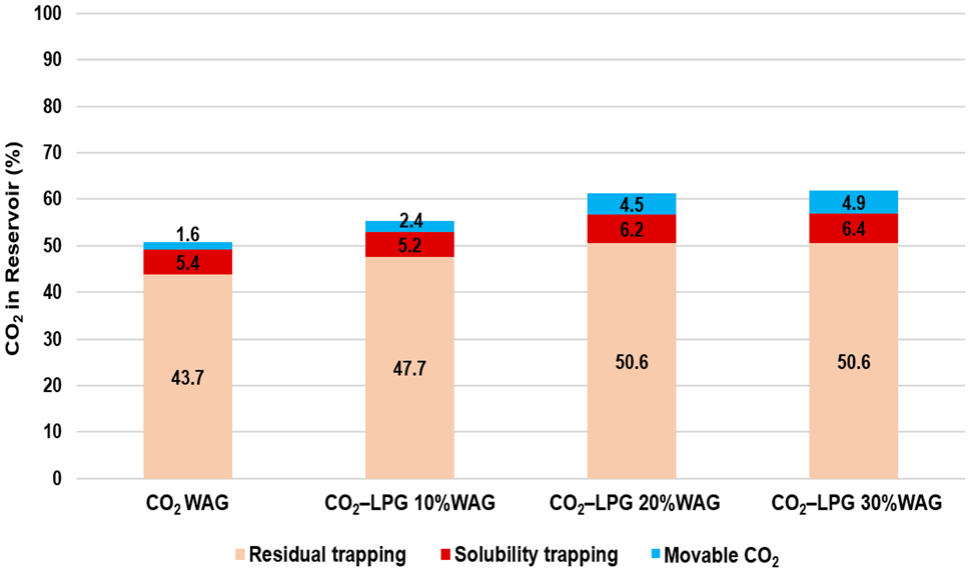
Remaining CO_2_ in the reservoir according to trapping mechanism with various LPG concentrations.

The ratio between the remaining CO_2_ in the reservoir and the injected CO_2_ was compared for different LPG concentration models (Fig. 15). The performance of residual trapping was enhanced until the added LPG concentration reached 20% because the increased wettability reduced the gas mobility. When the added LPG concentration was increased to 30%, the efficiency of residual trapping did not change, as the wettability had already reached its maximum and the gas mobility was not further reduced. Because greater asphaltene deposition increased the reservoir pressure, solubility trapping was enhanced. In contrast, the average reservoir pressure increased due to more-severe plugging effects, and the solubility trapping performance could be enhanced. In summary, the ratios of residual to injected CO_2_ were 50.7%, 55.3%, 61.3%, and 61.8% for cases with 100% CO_2_ and 10%, 20%, and 30% LPG additions, respectively. The efficiency of CCS tended to be enhanced as the concentration of LPG increased. Economic analysis should be performed for both EOR and CCS, because the improved CCS efficiency with LPG addition shows diminishing returns.

**Figure 16:**
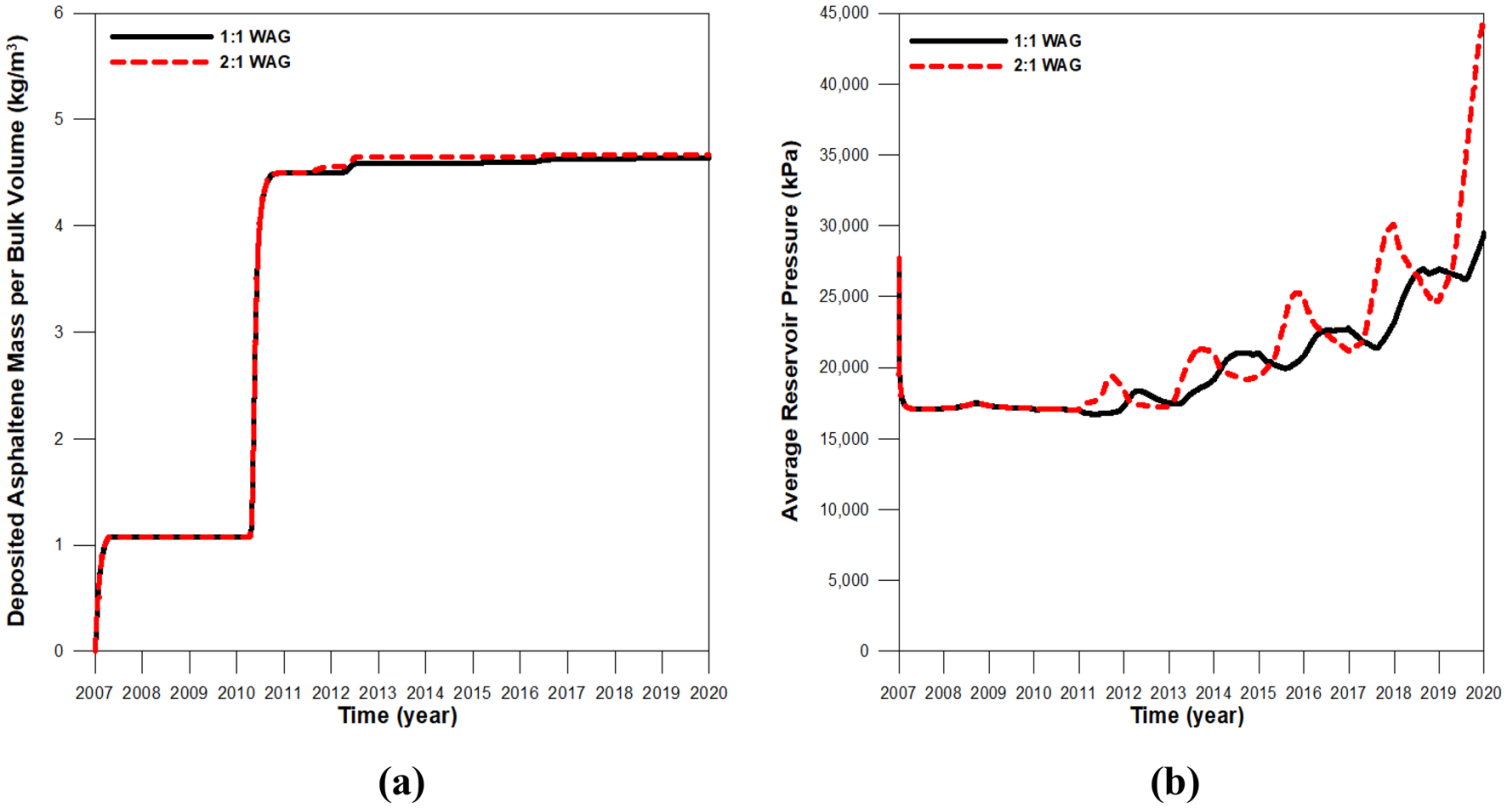
(**a**) The amount of asphaltene deposition per bulk volume in the middle of the reservoir and (**b**) the average reservoir pressure with different WAG ratios.

**Figure 17:**
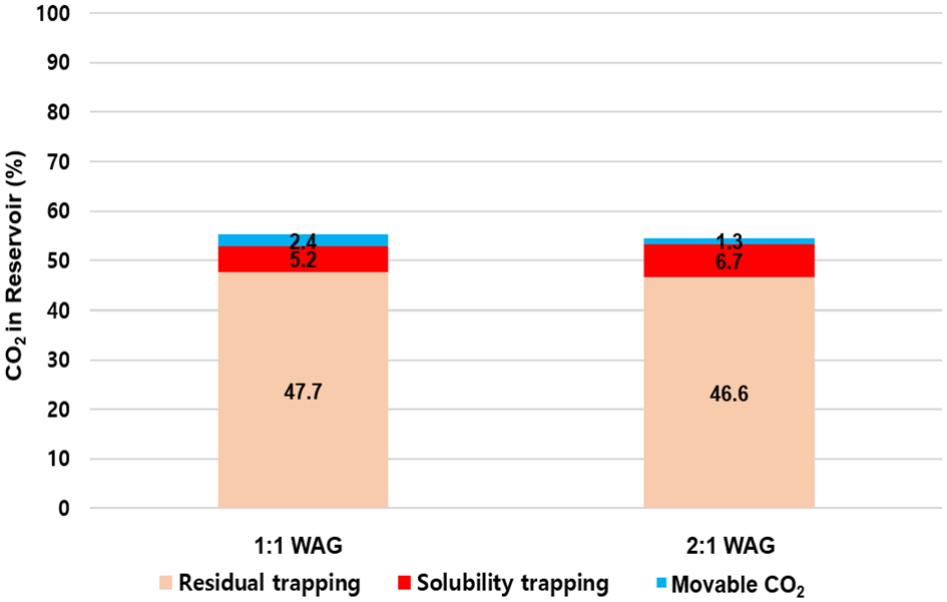
Remaining CO_2_ in the reservoir according to trapping mechanism with various WAG ratios.

**Figure 18:**
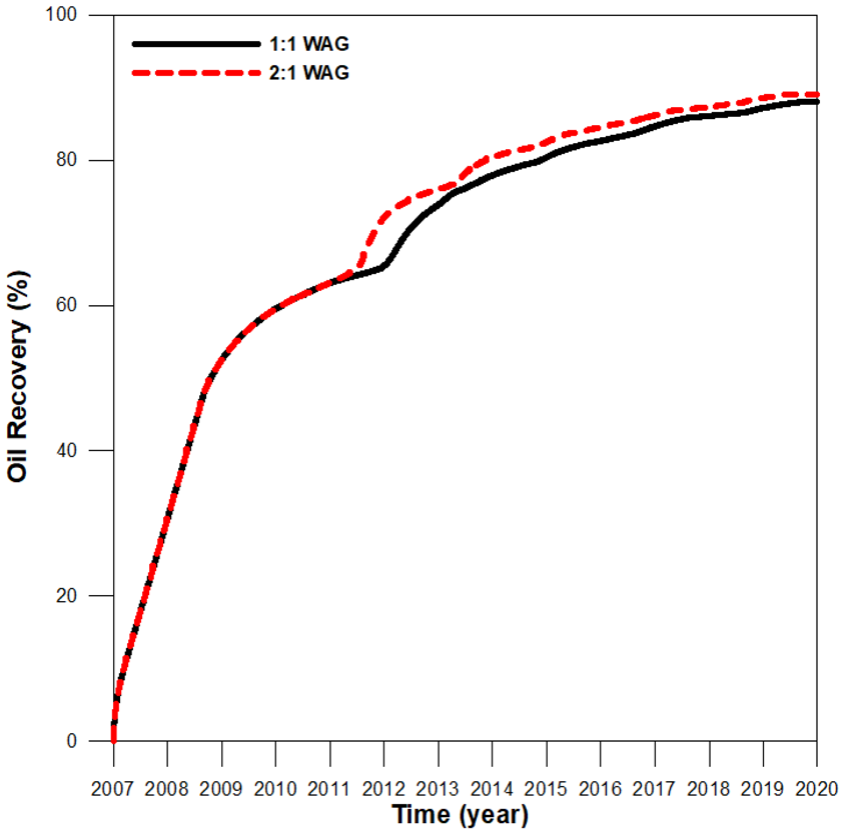
Oil recovery during CO_2_-LPG WAG injection in integrative models with different WAG ratios.

To detect the effect of the WAG ratio, simulations were also conducted for models with WAG ratios of 1:1 (the previous model) and 2:1 (the amount of water injection was double compared to the previous model) during CO_2_-LPG WAG. Because the same amount of gas was injected, the quantity of the deposited asphaltene was almost equal. However, the reservoir pressure increased due to greater water injection at a 2:1 WAG ratio (Fig. 16). The performance of CO_2_ residual trapping in the 2:1 WAG model was less than that of the 1:1 model because the duration of each inhibition period was longer. This caused CO_2_ to be expelled from trapped positions, despite the comparable amount of asphaltene deposition and wettability alteration. However, the higher reservoir pressure and greater water injection in the 2:1 WAG injection enhanced the performance of solubility trapping (Fig. 17). As a result, CCS performance did not change significantly, even though more water was injected in the 2:1 WAG injection. Increased water injection can therefore improve EOR performance with oil recovery by 1.1% (Fig. 18).

## Discussion

This study examined the effects of adding LPG to an CO_2_ stream in CCS-EOR with respect to asphaltene deposition using a compositional two-dimensional reservoir simulation. The effects of gravity and heterogeneity were ignored to focus on miscibility and damage to geological formations. We expect that the developed model can be expanded to a three-dimensional heterogeneous model that can be applied real field cases.

The significance and the speed of wettability alteration by asphaltene deposition remain controversial. Many studies, including this one, have assumed that wettability alteration depends on the amount of asphaltene deposition and the contact angle as determined by a computational method without experimental data^6,12,20,22^. This does not pose a logical problem, but it is necessary to select simulation parameters through experimentation to accurately evaluate the impact of asphaltene deposition on wettability alteration.

For field applications, an optimum CO_2_-LPG WAG design that considers economic feasibility, such as tax credits; CAPEX; OPEX; and the price of CO_2_, LPG, and oil, is necessary. Because these factors depend on reservoir characteristics, a site-specific design that incorporates economic feasibility is essential.

## Conclusions

An integrated model that incorporates a fluid model with a reservoir simulation under dynamic conditions was developed to investigate the effects of LPG on a coupled CCS-EOR process with asphaltene deposition. For the reservoir simulation of WAG-based CCS-EOR, a three-phase hysteresis model was applied to determine trapping phenomena under dynamic conditions. Formation damage from asphaltene deposition was incorporated into models of the CO_2_ and CO_2_-LPG WAG processes. The following conclusions can be drawn:

1. Because of the improved displacement efficiency caused by the increased oil viscosity, increased density, and IFT reduction, LPG enhances EOR performance during WAG injection. Reduction in oil density by LPG addition lowers the asphaltene solubility of oil, and CO_2_-LPG co-injection accelerates asphaltene precipitation and resulting formation damages. EOR performance of CO_2_-LPG WAG injection without asphaltene 18% higher than that with asphaltene, according to the model. The performance of CO_2_-LPG WAG injection can be overestimated if the formation damage is not considered.
2. An integrated model for CO_2_-LPG WAG injection with three-phase hysteresis was applied to the coupled CCS-EOR process. LPG lowers oil density by more than does CO_2_ WAG injection, and this accelerates asphaltene deposition. The residual trapping performance of CO_2_-LPG WAG injection can be improved compared with CO_2_ WAG injection. In contrast, more formation damages due to LPG addition lowers the solubility trapping performance compared with CO_2_ WAG injection, although LPG increases the reservoir pressure. Adding LPG improves total CCS performance by 9.1% and enhances oil recovery by 11% compared with CO_2_ WAG injection, and CO_2_-LPG co-injection enhances not only EOR but also CCS performance during CCS-EOR.
3. To apply the integrative model in various conditions, reservoir simulations were conducted with different LPG concentrations and WAG ratios. Although both EOR and CCS performances can be enhanced at higher LPG concentrations, the increment becomes smaller with more LPG. Therefore, economic evaluations should consider the price of LPG to determine the optimum LPG concentration. WAG ratios of 1:1 and 2:1 can also be compared to investigate the effect of the injection scheme with the same amount of gas. Although the amount of injected water with a 2:1 WAG ratio is much greater, long-term CCS and EOR performances do not change meaningfully. However, short-term oil recovery can be enhanced by up to 9.4% due to higher pressures and sweep efficiency. For optimum application of CCS-EOR in terms of LPG concentration and WAG ratios, an economic analysis that considers the price of LPG and water should be undertaken.
